# The valproate prescription pattern for female mental healthcare users of reproductive age

**DOI:** 10.4102/sajpsychiatry.v30i0.2158

**Published:** 2024-03-26

**Authors:** Phumla S. Gasa, Andrew Tomita, Vidette Juby, Saeeda Paruk

**Affiliations:** 1 Department of Psychiatry, College of Health Sciences, University of KwaZulu-Natal, Durban, South Africa; 2 KwaZulu-Natal Research Innovation and Sequencing Platform (KRISP), College of Health Sciences, University of KwaZulu-Natal, Durban, South Africa; 3 Centre for Rural Health, School of Nursing and Public Health, College of Health Sciences, University of KwaZulu-Natal, Durban, South Africa

**Keywords:** valproate, prescription, women of reproductive age, South Africa, mental health care user

## Abstract

**Background:**

Sodium valproate (valproate) that is used both as an anti-epileptic and a mood stabiliser is teratogenic in pregnancy. A Dear Health Care Professional Letter (DHCPL) issued in December 2015 recommended the avoidance of sodium valproate prescription in women of childbearing age (WOCBA) and pregnant women.

**Aim:**

This study aimed to describe the prescription pattern of valproate in female mental healthcare users (MHCUs).

**Setting:**

Regional hospital psychiatry department in King Dinizulu Hospital Complex, Durban, KwaZulu-Natal.

**Methods:**

This was a descriptive, retrospective chart review of female in- and out-patient aged 12–55 years who were receiving a valproate prescription for mental illness between 01 January 2018 and 31 December 2020.

**Results:**

Of the 158 females who received valproate during the study period, 15 (9.5%) had it tapered off while 143 (90.5%) were continued. Only 19% of all the patients had documented counselling regarding valproate, 19 (12%) had documented contraceptive use, and six (3.8%) continued its use at any point during pregnancy. The most frequently prescribed dose range was 800 mg – 1499 mg/day (*n* = 111, 70.7%) and the most common psychiatric indication was a psychotic disorder.

**Conclusion:**

This study showed that prescription of valproate in female MHCUs still occurs in practice in a referral centre in South Africa despite the guidelines outlining management of those of reproductive age on valproate. The prescription pattern and monitoring of valproate were poorly documented in relation to the guideline.

**Contribution:**

This study highlights the lack of adherence to recommendations regarding the prescription of valproate in WOCBA and the need for improved documentation of the indications, consent and counselling.

## Introduction

The drug valproate is used both as an antiepileptic and a mood stabiliser, with increasing use for conditions other than epilepsy,^1^ its main use in the field of psychiatry being for bipolar disorder.^2,3^ It has also been used in other psychiatric disorders to augment the treatment of schizophrenia and borderline personality disorder. As a monotherapy, it is as efficacious as antipsychotics and lithium as a mood stabiliser, but in combined use with antipsychotics has more efficacy than either drugs alone.^4^ Valproate is hence a commonly prescribed mood stabiliser in bipolar disorders in monotherapy or in combination regimens.^5^

In utero exposure to valproate, particularly in the first trimester, can result in foetal valproate syndrome (FVS), which includes dysmorphic features and congenital malformations (10% probability), namely neural tube defects, for example, spina bifida, cardiovascular abnormalities and developmental delays.^3,6,7^ The long-term effects include a higher risk for the development of neuro-developmental disorders (30% – 40% probability), such as autism and learning difficulties. A reduction in intelligence quotient (IQ) has also been reported and seen to be linked with higher doses (above 800 mg per day),^8,9^ with those of 1500 mg per day or higher posing even greater risk.^8,10^

Besides the risk to the foetus or unborn child, valproate has adverse health risks in general, with side effects affecting fertility, specific to females. Polycystic ovarian syndrome (PCOS) is likely the most common endocrine disorder affecting female fertility in those of reproductive age. Polycystic ovarian syndrome and its features (polycystic ovaries, hyperandrogenism and menstrual abnormalities) were more prevalent in females taking valproate compared to those treated with other antiepileptic drugs for epilepsy.^11,12^ Valproate use is also associated with weight gain and insulin resistance, which increases metabolic and cardiovascular risk, as well as the risk for PCOS.^13^

In December 2015, a Dear Health Care Professional Letter (DHCPL) was issued and included recommendations for the avoidance of sodium valproate prescription in women of childbearing age (WOCBA) and pregnant women.^14,15^ The Medicines and Healthcare products Regulatory Agency (MHRA) guidelines released in April 2018 restricted the use of valproate in women of childbearing potential unless they were on a pregnancy prevention programme (PPP).^10^ In 2019, the MHRA guidelines formed the cornerstone for the National Institute for Health and Care Excellence (NICE) guidelines, echoing the caution of valproate in females of reproductive age unless they were on a PPP. The PPP entails that the user is informed about the risks of valproate and on highly effective contraception and has to complete an annual risk acknowledgement form (ARAF) regarding this.^3,6,10^ In the South African context, the South African Health Products Regulatory Authority (SAHPRA) and SANOFI South Africa published guidelines for the use of valproate in females of child bearing age.^16,17^

Other safe prescribing bodies also concurred with these guidelines and the potential teratogenic effects of valproate such as the British National Formulary, European Pharmacovigilance Risk Assessment Committee (PRAC), Coordination Group for Mutual Recognition and Decentralised Procedures-Human (CMDh), and the International College of Neuro-Psychopharmacology treatment guidelines for Bipolar Disorder in adults (CINP-BD-2017)).^18,19,20^ In addition, in September 2018, the South African Department of Health issued a notice ‘Sodium Valproate Unsafe in Pregnancy’^21^ and the SAHPRA letter was updated in 2019, stating that valproate should be avoided in pregnancy and in women of childbearing potential.^7,16^

Despite these guidelines, the use of valproate in females of childbearing age, particularly in the treatment of bipolar disorder, has continued to increase in some studies and remains unchanged in others.^2,7,10,22^

A recent South African study showed increasing awareness but a lack of proper understanding of valproate risks among patients, probably because of healthcare professionals providing inadequate information during counselling.^10^ The increase in awareness is a starting point in the South African context, although research is limited.

In the international context, a study in the United Kingdom (UK) showed a decline in awareness of risks associated with valproate by the users despite the new guidelines, and similar to the South African context, users received inadequate counselling by the health care professionals and showed sub-optimal understanding of risks.^23^ More recent international studies making comparisons before and after the MHRA guideline noticed a decrease in valproate exposed pregnancies and valproate prescriptions in the population of interest, and an increase in preconception advice and use of contraception in those using valproate.^2,3,6^ This study therefore aimed to determine the prescription pattern and adherence to the prescriber guidelines for the use of sodium valproate among females of reproductive age with mental illness at a psychiatric hospital in KwaZulu-Natal (KZN) province, South Africa.

## Research methods and design

### Study design, setting, participants and procedure

This quantitative descriptive, retrospective chart review of in- and out-patient females was conducted at a psychiatric service in KZN province, South Africa. The study was carried out at a public sector tertiary hospital psychiatric department that provides specialised psychiatry services to the eThekwini Municipality. Access to specialised care (tertiary) starts with a referral from primary healthcare facilities, to district, regional, then tertiary psychiatry units.

The study population consisted of female patients aged 12–55 years with a valproate prescription who attended the psychiatric department from 01 January 2018 to 31 December 2020 and for whom any *Diagnostic and Statistical Manual of Mental Disorders, Fifth Edition* (DSM 5) psychiatric diagnosis was available. All data were anonymised.

### Measures

The structured data sheet consisted of socio-demographic and clinical questions based on the literature. Data collected included age, level of education, marital and employment status, primary DSM-5 diagnosis, comorbid medical and psychiatric diagnoses, metabolic risk (glucose level, presence of hypertension or diabetes), pre-existing or new sodium valproate prescription, as well as monitoring sodium valproate levels, full blood count (FBC), liver function test (LFT), urea and electrolytes, valproate dose, documentation of signed informed consent for valproate use and documented contraceptive use (included all oral, surgical or barrier methods) in clinical notes.^24,25^

### Statistical methods

The data were extracted from the patient files by a medical doctor with clinical psychiatry experience, manually recorded on the data forms and captured electronically onto the academic software tool, Redcap. Descriptive statistics, such as frequencies, percentages, mean and standard deviation, were used to summarise the data. The data collected were analysed with SPSS version 28.0 (IBM Corp, Armonk, NY, USA) and Stata version 16.0 (StataCorp, College Station, TX, USA). Categorical data were presented as frequencies and percentages. Descriptive statistics (mean and standard deviation) were used to describe the continuous data that were collected.

### Ethical considerations

The study was approved by the Biomedical Research Ethics Committee (BREC) (reference number is BREC/00002649/202) of the University of KZM, the KZN Department of Health and the hospital. Data were obtained from clinical records available at the hospital. Permission to conduct this study was sought from the hospital and Department of Health (DOH). All data with potential identifiers were removed and replaced with codes to ensure anonymity of participants.

## Results

### Socio-demographic profile

A total of 158 files of females who met the inclusion criteria were included. Some of the findings regarding the socio-demographic profile included the following: 69 patients receiving valproate were of Indian race (*n* = 69, 43%), with the highest age group for valproate prescription being between 40 and 49 years (*n* = 50, 31.6%), single or divorced or widowed (*n* = 122, 78.7%), unemployed (*n* = 111, 70.3%) and had tertiary education (*n* = 27, 19.1%) (Table 1).

**TABLE 1:** Sociodemographic and clinical characteristics (*N* = 158).

Variable	Category	*n*	%
Race	Black people	61	38.6
	Indian people	69	43.7
	Mixed race people	19	12
	White people	9	5.7
Age category (years)	12–29	31	19.9
	30–39	42	26.9
	40–49	50	32.1
	50+	35	22.1
Marital status	Married	33	21.3
	Single, widowed or divorced	122	78.7
	Missing	3	1.8
Education	Remedial	6	4.3
	Mainstream	108	76.6
	Tertiary	27	19.1
Employment	Employed	28	17.7
	Unemployed	108	70.3
	Disability grant	27	12
Admission status	Inpatient	10	6.3
	Outpatient	148	93.6
Number of admissions	0	82	52.2
	1	59	37.6
	2	14	8.9
	3	2	1.3
Psychiatric diagnosis	ASD/ID	10	6.4
	Bipolar disorder	58	37.2
	Psychotic disorder	69	44.2
	Depressive/anxiety disorder	19	12.2
HIV status	Negative	129	81.6
	Positive	20	12.7
	Unknown	9	5.7
Medical comorbidities	Hypertension	34	21.5
	Diabetes	19	12
	Epilepsy	19	12
	Traumatic brain injury	2	1.3
	Tuberculosis	2	1.3
Current substance use	Tobacco	58	36.7
	Cocaine	2	2.5
	Cannabis	17	10.7
	Alcohol	37	23.4
Sexual activity	Sexually active	19	12
	Not sexually active	5	3.2
	Undocumented	134	84.8

ASD/ID, autism spectrum disorder/intellectual disability.

In terms of their clinical profiles, the majority were outpatients during the study period (*n* = 148, 93.6%), with 52.53% (*n* = 83) having never been admitted, 37.34% (*n* = 59) having had one admission, 8.86% (*n* = 14) with two admissions and 1.27% (*n* = 2) with three admissions. The most common indication for valproate prescription was a primary psychotic disorder (*n* = 69, 44.2%), then bipolar disorders (*n* = 58, 37.2%) and depressive/anxiety disorders (*n* = 19, 12.2%). The documented human immunodeficiency virus (HIV) status was positive in 20 (12.7%) and negative in 129 (81.6%) participants. Their medical comorbidities included hypertension (*n* = 34, 21.5%), diabetes, epilepsy, head injury and tuberculosis, which accounted for (*n* = 19, 12%), 19 (12.5%), 2 (1.3%) and 2 (1.3%), respectively (Table 1).

### Valproate prescribing patterns

The majority of valproate prescriptions were initiated at tertiary level health facilities (*n* = 101, 64.74%), followed by the district (*n* = 28, 17.75%) and regional levels (*n* = 15, 9.62%), with few being prescribed by the private sector (*n* = 11, 7.05%) and primary healthcare facilities (*n* = 1, 0.64%). The valproate daily dose ranged above 700 mg – 1500 mg (*n* = 111, 70.3%), to 1500 mg – 2000 mg (*n* = 33, 20.9%) and ≤700 mg (*n* = 12, 7.6%), with only one person (0.6%) being on a daily dose of above 2000 mg (Table 2).

**TABLE 2:** Valproate prescription characteristics (*N* = 158).

Variable	Category	*n*	%
Continued valproate use	Yes	137	86.7
	No	21	13.3
Indication	Primary psychiatric diagnosis	143	90.5
	Medical diagnosis	4	2.5
	Both	11	7
Level of facility initiating valproate	Tertiary	101	64.7
	Regional	15	9.6
	District	28	17.9
	Primary	1	0.6
	Private care	11	7.1
Daily dose	≤ 700 mg	12	7.6
	> 700 mg–1500 mg	111	70.7
	> 1500 mg – 2000 mg	33	21.0
	> 2000 mg	1	0.6
Recent valproate blood level	Normal	64	41.3
	High	2	1.3
	Low	21	13.5
	Undocumented	68	43.9
Valproate exposure in pregnancy in study period	Yes	6	3.8
	No	18	11.4
	Undocumented	134	84.8
Contraception use	Yes	19	12
	No	38	24.1
	Undocumented	101	63.9
Documented counselling and/or informed consent	Yes	30	19
	No	128	81

### Documented informed consent and contraceptive prescription

Valproate prescription was stopped during the study period in 21 (13.3%) females, while 137 (86.7%) had continued valproate prescriptions. Valproate use during any point in pregnancy was recorded for (*n* = 6, 3.8%). A total of 134 (84.8%) files had not recorded the pregnancy status prior to valproate administration or during the course of valproate treatment and 18 (11.4%) files had a negative pregnancy test documented prior to administration. Six patients were subsequently recorded as becoming pregnant while on valproate. Contraceptive use was documented for 24.1% (*n* = 38, 24.1%) of the females, while counselling on and consent for valproate use was documented for 30 (19.9%) females (Table 2).

### Clinical monitoring

Blood test monitoring of valproate levels was documented in 36 (22.8%) females, with the required 6 months and annual monitoring being poorly documented (Table 3).

**TABLE 3:** Clinical monitoring.

Investigation	Baseline		6 months		Annual	
	*n*	%	*n*	%	*n*	%
FBC	116	73.4	18	11.4	73	46.2
LFT	110	69.6	14	8.9	10	6.3
U&E	117	75.1	12	7.6	71	44.9
Cholesterol	13	8.2	4	2.5	29	18.4
Glucose	26	16.5	12	7.6	30	19
Valproate	36	22.8	18	11.4	54	34.2
Pregnancy test	16	10.1	1	0.6	0	0

FBC, full blood count; LFT, liver function test; U&E, urea and electrolytes.

Note: Valproate: 346 umol/L – 693 umol/L.

### Co-prescriptions

Of the 158 files, 157 (84.2%) had co-prescription of valproate with other medications (Table 4), of whom two (1.3%) were prescribed lithium, while 17 (10.8%) had other mood stabilisers. First generation antipsychotics accounted for 39 co-prescriptions (24.7%) and co-prescription of second-generation antipsychotics was noticed in 74 females (46.8%).

**TABLE 4:** Co-prescription of other medications.

Variable	*n*	%
First generation antipsychotic	39	24.7
Second generation antipsychotic	74	46.8
Lithium	2	1.3
Other antiepileptics	1	0.6
Other mood stabilisers	17	10.8

## Discussion

The study aimed to determine the prescription pattern and adherence to the prescriber guidelines for the use of sodium valproate among females of childbearing age with mental illness at a psychiatric hospital in KZN province, South Africa.

### Socio-demographic profile

A total of 158 females were prescribed valproate, the key findings being that valproate still remains prescribed in WOCBA, with most receiving more than 800 mg/day for psychiatric disorders, the files indicating poor documentation of informed consent, contraception use and monitoring for side effects.

As a cross-sectional study, the authors could not assess if valproate prescriptions had decreased in number over time, but its continued use in 158 women (in an outpatient clinic seeing 350–375 female mental healthcare users [MHCUs] per month) 5 years after the DHCPL memorandum remains concerning. This may be because of patients being initiated prior to the current guideline. Of note, valproate use was discontinued in 21 females during the study period. The majority of women continued on valproate were outpatients and had no admissions during the study period (Table 1), which might suggest a fairly clinically stable population on valproate who may have not been planning to have children, but this was not well documented. This finding could also highlight that more complex cases being reviewed in a tertiary level setting providing appropriate specialised management required more than one mood stabiliser. However, the possible adverse effects that could result from valproate prescription and precautionary measures should have been discussed with the patient and documented on the outpatient review, as per the MHRA guidelines, which state that valproate should not be used in females of childbearing potential unless there is no other suitable alternative.^10^ The study’s findings are consistent with other international audits, which report that, despite the manufacturers guidelines, valproate is still prescribed in females of childbearing age.^26^

### Valproate prescription pattern

The main indication for valproate use in the study population was for psychiatric disorders, with psychotic disorders (*n* = 69, 44.2%) being the main indication, which differs from the literature, where the main psychiatric indication was bipolar disorders.^2,3^ In this study, the use of valproate may have been because of the complexity of the patients at a specialised psychiatry service who required antipsychotic treatment and augmentation with a mood stabiliser.^27^

The majority of females had valproate prescriptions that were greater than 800 mg/day; this is concerning as the development of FVS could be dose dependent. Teratogenic risk with valproate is seen to be greatest with daily doses above 1000 mg (other studies state daily doses above 1500 mg).^8,10,26^ This finding is consistent with the literature, and while Paton and colleagues showed that females were prescribed doses lower than males, these doses were still greater than 600 mg/day.^26^

### Documented informed consent and contraceptive prescription

The recommendations and guidelines regarding valproate use state that adequate information needs to be given regarding the use of valproate and the implications.^10^ Females who remain on valproate therefore need to be on a mandatory PPP, be offered effective contraception and are required to complete an ARAF regarding this information.^10,18,19^ This study showed that inadequate documentation and counselling was given about valproate use, with only 30 (19%) clinical records having recorded its occurrence. Sexual activity was undocumented in 84.8% of the study population, while the majority (*n* = 101, 63.9%) had no documentation of contraception use status. Of those with documented contraceptive status, 66.6% were not on contraception despite valproate use and guideline recommendations. Possible explanations for this major deficit in clinical care documentation could be inadequate healthcare worker knowledge on valproate prescription, the complexity of patient profiles (the majority were on more than one drug)^28^ or limited alternative therapeutic options in public sector facilities. Studies conducted in settings that were not resource constrained also showed poor reporting of adherence to guidelines.^29,30,31^

Valproate exposure during pregnancy has been shown to have teratogenic effects and should be discontinued. The PPP indicates that females of childbearing potential should have a baseline pregnancy test before treatment initiation, and repeated during the course of treatment with valproate.^19^ In this study, six females (3.8%) were documented to have had continued valproate exposure during a pregnancy, while other studies reported a decline in valproate exposed pregnancies after the release of the MHRA guidelines.^2,6^ The cross-sectional design does not enable inferences to be made about trends in this setting, but is consistent with the literature of low numbers of pregnant women being continued on valproate.^29^

### Clinical monitoring

Besides teratogenic and neuro-developmental risks associated with valproate,^10,23,26,28^ the drug is associated with other side effects that directly affect the female, such as weight gain, PCOS and hyperandrogenism.^23^ Risk of hepatotoxicity has also been noticed, with the need to monitor for this being outlined in other research.^32,33,34^ For these reasons, baseline and follow-up monitoring of parameters, such as serum cholesterol and glucose, while on sodium valproate is important, as well as other baseline bloods, such as LFTs.^17,24^ In this study, baseline and follow-up haematological monitoring of other adverse effects associated with valproate, such as hepatotoxicity, were poorly documented. The valproate levels (documented in 36 files), cholesterol (documented in 13 files) and glucose (documented in six files) were again in keeping with poor documentation.

### Co-prescriptions

Finally, in this study, 84.2% (*n* = 133) of the 158 females were receiving a combination prescription, and included co-prescription with other mood stabilisers and antipsychotic medications (mainly second generation antipsychotics), suggesting that valproate was used for augmentation of antipsychotics for psychotic illnesses^28^ or for bipolar illness that required addition of antipsychotics in acute phase. Previous studies found that in the treatment of acute mania, valproate monotherapy was as efficacious as antipsychotics and lithium, but that in combined use with antipsychotics, had greater efficacy than either drugs alone.^4^ While the results of this study were in keeping with other research, which showed that valproate was commonly prescribed in combination with other drugs, its use in this sub-population needs careful review and monitoring, which appears to have been lacking.

### Recommendations

The findings in this study showed that the SAHPRA guidelines set out in 2018 for the use of sodium valproate were not fully adhered to in this study population. There was very poor documentation of information and counselling on its use and effects on pregnancies, placing females on a PPP and finding safer alternative medications in its place. This risk should be reason enough to make it mandatory for healthcare professionals to adhere to the correct prescription of valproate in the female population, specifically in mental health patients, where valproate is commonly prescribed to a very vulnerable group. A checklist with mandatory questions and investigations, as well as signed acknowledgement of information and counselling about valproate, should be completed at baseline and the biannual or annual reviews of patients to ensure that the guidelines are adhered to. Relevant information about valproate needs to be provided to prescribers on a regular basis so that they are familiar with the serious effects of valproate in an effort to create better awareness and therefore enable them to impart this to their patients.

### Limitations

A number of limitations may have affected the findings. The first being that a hospital-based study may introduce sample bias and limit generalisability to community samples. With the study being a retrospective review and its cross-sectional nature, could lead to changes being missed over time, and issues related to the quality of record keeping not being addressed. Foetal valproate syndrome might be dependent on valproate dose; the mean dose that was prescribed in this population was not studied as reference was only made to dose ranges. The authors also did note if valproate dose was in the process of being weaned or cross titrated during its use in the study period. Socio-demographic data were collected from the start of the study period and did not account for changes in some of the variables during the study period. The World Health Organization (WHO) found that women of reproductive age is up to 49 years, while the Centers for Disease Control and Prevention contraceptive guidance for healthcare providers observe that the age of definitive loss of natural fertility is 41 years but can range up to age 51 years.^35,36^ The inclusion of females up to the age of 55 in this study could be seen as questionable. There is also no control group and includes women up to age 55 who may have completed families and hence risk of teratogenicity was less concerning. Pregnancy outcomes following valproate exposure would have been valuable data to study, but the pregnancy outcomes of the females that were exposed to valproate at any point during their pregnancy were not looked at in this study.

## Conclusion

This study showed that prescription of valproate in female MHCUs still occurs in practice in South Africa despite the guidelines outlining management of those of reproductive age on valproate. Other aspects of the guidelines regarding counselling on valproate use and contraception use, were poorly documented, as was overall monitoring. There needs to be ongoing efforts to remind clinicians about the conditions under which valproate can be prescribed and for improved documentation of the clinical factors that are important to ensure optimal clinical care.
